# Editorial: Insights in methods and model organisms: 2021

**DOI:** 10.3389/fnmol.2022.1104424

**Published:** 2022-12-06

**Authors:** Stephan C. F. Neuhauss

**Affiliations:** Department of Molecular Life Sciences, University of Zurich, Zurich, Switzerland

**Keywords:** gene editing, neurobehavior tests, mouse, zebrafish, biomarker, Alzheimer's disease, autism spectrum disorder, alcohol use disorder

For this Research Topic, we solicited forward-looking contributions on current and prospective use of methodology and models organisms in the field of molecular neuroscience. Indeed, we received a thought-provoking mix of review and original research articles that are briefly summarized in this editorial.

The review by Baratta et al. is written very much the same vein of this Research Topic in reporting on advances in genomic and behavioral analysis to understand normal and pathological brain function. Both approaches are the focus of intense research activities, taking advantage of both new instrumentation and increasingly more important data analysis tools. The complex and exciting relationship between genes and behavior necessitates such multipronged experimental approaches.

Enormous advances in imaging technology (including sample preparation and microcopy) and genetic engineering technologies have revolutionized the neurosciences. This is particularly apparent in the impressive number of studies published in the last few years using techniques that are ever more sophisticated to label specific cell types and their connections in the nervous system of mice. The review article by Arias et al. presents a much-needed guide to recent advances in the development of fluorescent transgenic mouse models with an eye to their use in understanding disease mechanisms.

Goel and Ploski discuss in their review the merits of short hairpin RNA-mediated (hRNA) gene silencing in light of the more recent gene editing tools provided by the CRISPR/Cas system. A particular concern is the chance of off-target effects of shRNA that may limit its utility in comparison to alternative CRISPR/Cas approaches.

In an original research article, Deng et al. describe a patient with a *de novo* non-sense mutation in the BRSK2 gene exhibiting characteristics of autism spectrum disorder (ASD). They went on to create a zebrafish mutant model using genome editing. BRSK2 deficient fish displayed neurodevelopmental deficits consistent with altered expression level of neurogenesis-related genes. Behaviorally these fish display impaired social preferences and shoaling behavior, establishing a new ASD animal model.

Alzheimer's disease (AD) is a recognized public health issue commanding a huge amount of research funding, predominantly using mice to model the disease. Yokoyama et al. discuss a variety of mouse models, including transgenic, knock-in, and injection based model, aiming to recapitulate aspects of the pathophysiological processes in AD. Other mouse models based on neuroinflammation and the involvement if microglia are also discussed.

Tello et al. address another approach to the study of AD in their review by reporting on recent advances on drug discovery using the fruit fly *Drosophila melanogaster*, a prominent invertebrate model for drug discovery. They argue that the most useful approach is to combine multiple screening methods such as morphological, behavioral and biomolecular analyses.

It is an important quest of diagnostic medical research to identify molecular biomarkers that are able to assist in diagnosis, patient stratification and prediction of disease outcome. Studies on the highly prevalent alcohol use disorder (AUD) are no exception. Ferguson et al. describe the latest research on using transcriptomic data and their analysis using artificial intelligence to identify molecular biomarker aiming at improving clinical management of AUD.

The reproducibility of any scientific experiment is one of the basic tenants of science. Ideally, any publication should provide all the information needed to repeat the describe experiments in any laboratory yielding comparable results. Much has been written about the current reproducibility crisis in Science with some of the discussions centering on the detailed description of experimental procedures. This is particularly true for behavioral experiments with its abundant ambient parameters affected outcome. Neuwirth et al. address the reporting of light conditions in their systematic review in publications on a number of standard behavioral neuroscience tests. They found that only a minority of reports state the exact lighting conditions under which the tests have been performed. The authors provide suggestions on how the behavioral neuroscience community should address this untenable situation helping efforts to booster validity of behavioral experiments.

## Author contributions

The author confirms being the sole contributor of this work and has approved it for publication.

